# Infant mortality among native-born children of immigrants in France, 2008–17: results from a socio-demographic panel survey

**DOI:** 10.1093/eurpub/ckaa186

**Published:** 2020-11-30

**Authors:** Matthew Wallace, Myriam Khlat, Michel Guillot

**Affiliations:** 1 Stockholm University Demography Unit (SUDA), Sociology Department, Stockholm University, Stockholm, Sweden; 2 Mortality, Health and Epidemiology (URO5), French Institute for Demographic Studies (INED), Paris, France; 3 Population Studies Center, University of Pennsylvania, Philadelphia, PA, USA

## Abstract

**Background:**

Within Europe, France stands out as a major country that lacks recent and reliable evidence on how infant mortality levels vary among the native-born children of immigrants compared with the native-born children of two parents born in France.

**Methods:**

We used a nationally representative socio-demographic panel consisting of 296 400 births and 980 infant deaths for the period 2008–17. Children of immigrants were defined as being born to at least one parent born abroad and their infant mortality was compared with that of children born to two parents born in France. We first calculated infant mortality rates per 1000 live births. Then, using multi-level logit models, we calculated odds ratios of infant mortality in a series of models adjusting progressively for parental origins (M1), core demographic factors (M2), father's socio-professional category (M3) and area-level urbanicity and deprivation score (M4).

**Results:**

We documented a substantial amount of excess infant mortality among those children born to at least one parent from Eastern Europe, Northern Africa, Western Africa, Other Sub-Saharan Africa and the Americas, with variation among specific origin countries belonging to these groups. In most of these cases, the excess infant mortality levels persisted after adjusting for all individual-level and area-level factors.

**Conclusions:**

Our findings, which can directly inform national public health policy, reaffirm the persistence of longstanding inequality in infant mortality according to parental origins in France and add to a growing body of evidence documenting excess infant mortality among the children of immigrants in Europe.

## Introduction

The origin composition of births in France has transformed in the last few decades. Births to at least one foreign-born parent now contribute a third of all annual births—one of the largest shares in all of Europe^1^—and the majority of this share have a parent, or parents, with origins outside of the EU.^2^ Despite this growing diversity, France stands out as a European country that lacks up-to-date evidence on how infant mortality levels vary according to parental origins. This is perhaps due to a national citizenship model that negates using such criteria to classify people and treat them as distinct groups in order to promote equality—a principle enshrined in public health care services.^3^ Despite this, evidence from government reports suggests that health inequalities are readily apparent from birth among the native-born children of immigrants, notably in the lower survival chances of children with parents from Africa.^4–6^ However, these reports are now dated, and their aim was simply to describe differentials in infant mortality according to the origins of the parents.

Thus, the objectives of this study are to contribute up-to-date findings on ‘how’ and ‘why’ infant mortality varies among the native-born children of immigrants in France for a recent period, by describing initial mortality levels according to detailed parental origins and by assessing the explanatory role of a range of relevant socio-demographic predictors. We place our work within a European context in which elevated infant mortality in the children of parents with non-EU origins is common.^7–21^ We add to the evidence by providing recent findings for a major European country. We extend it by contributing one of the first studies to adopt a multi-level framework examining the role of proximal (parental) and distal (socio-geographic) factors in variation in infant mortality according to parental origins. The lack of explanatory power of proximal factors in poor immigrant birth outcomes has re-focused attention on the role of more distal factors,^22–24^ which can affect child health ‘directly’ through enabling parental access to resources and services^22^ and ‘indirectly’ through their effect on parental socio-economic position. Considering prior evidence, we expect to find elevated infant mortality among the native-born children of immigrants in France.

## Methods

### Data

We used the French Permanent Demographic Sample (EDP), France’s largest socio-demographic panel and representative 4% sample of the population. The EDP contains individual-level anonymized records on life events from civil registers, censuses and geographic data at a fine-grained level of administrative division in France—the commune—facilitating multi-level analysis. Eligibility for the sample is based upon date of birth, being born on one of 16 specific days in the calendar year. New members enter the sample by being born in, or moving, to France and exit the sample by death or emigration. For our study, we only need to link the birth and death files between 1 January 2008 and 31 December 2016. Thus, our analysis is representative of all births in France—including documented and undocumented immigrants—and is not selective upon, say, requiring an address in order to be able to complete a census form. The EDP is entirely socio-demographic and does not contain individual-level health data of any kind.

### Study parameters

Eligibility for the study is based upon being born in mainland France between 1 January 2008 and 31 December 2016. Mainland France refers to the area of the French Republic that is geographically in Europe and not overseas France (i.e. French Guiana, Guadeloupe, Martinique, Mayotte and Réunion). All children born in overseas France and all children born in mainland France to a parent—or parents—born in overseas France, are excluded from the analyses. The outcome variable is binary and indicates whether each infant born alive survived to his or her first birthday. The variable was derived from calculating the exact age at death, itself derived from the date of death (from the death file) minus the date of birth (from the birth file).

The exposure variable is parental region of origin. The native-born children of immigrants were defined as children born in mainland France to at least one parent born abroad. The reference group were defined as children born in mainland France to two parents born in mainland France. To categorize children according to their parental origins, we grouped countries into eight regional categories using the United Nations M.49 classification. For the 3600 cases (4.4% of the native-born children of immigrants) where parents were born in different regions, we assigned children according to mother’s region of origin. The composition of these groups can be found in online Supplementary table S3, which shows the main countries within each of these regional groups.

Alongside the parental region of origin, we adjusted for several other individual-level predictor variables that included the sex of the child (male vs. female), the year of birth (categorized into single years from 2008 to 2016), the age of the mother (categorized into 5-year bands ranging from 15–19 to 40–49), single vs. multiple birth (1 vs. 2+), and father’s socio-professional category (SPC; grouped as executive and intellectual professions, intermediate professions, office workers, manual workers, farmers and self-employed including craftsmen, small business leaders and shopkeepers). All of the predictor variables, including the parental country of birth, were derived directly from data from the birth certificate in the EDP.

At the commune-level, we adjusted for the French Deprivation Index (FDI), a composite indicator of neighbourhood deprivation based upon average household income, percentage high school graduates in the population aged ≥15 years, percentage of blue-collar workers in the active population and the unemployment rate. The index was originally designed to be representative for the whole of France and to take urban-rural comparability issues into account.^25^^,^^26^ The index is readily divided into quintiles of least, less, middle, more and most deprived. As a measure of urbanicity, we adjusted for the size of the urban unit, a spatial measure defined by the National Institute of Statistics and Economic Studies (INSEE). This measure classifies areas into rural areas of less than 2000 residents and urban areas of 2000–19 999, 20 000–199 999, 200 000–1 999 999, and 2 000 000+ (the Paris region) residents. In both cases, we merged these data into the EDP externally, attaching a category to each birth based upon the mother’s place of residence as registered on the birth certificate for the middle of the observation period, 2013.

The choice of the final individual-level predictors was based upon an initial exploratory data analysis of available variables in the EDP. For example, the birth file also contained information on birth order and mother’s SPC. We initially considered these variables as important predictors that might help to explain group differences. However, birth order was missing for 40% of births and mother’s SPC for 30% of births (with higher levels among immigrant groups); these births also had higher infant mortality and so we decided not to use them in the analysis. The inclusion of several other predictors was explored and dismissed due to moderate levels of missingness (>20%; birth interval and parental living arrangements) or a lack of association with infant mortality (parental civil status). At the area-level, we considered the % of the commune: in poverty, low educated, unemployed and/or foreign-born. However, we found the level of urbanicity and commune deprivation score to have the most consistent associations with infant mortality.

Due to the high quality of the data and the completeness of birth and death certificates, we only have a small number of excluded cases. Of the 303 260 births potentially eligible for inclusion in the analyses, we dropped five cases in which no country of birth was specified on the birth certificate. For parental country of birth, we dropped 6500 (2%) cases in which we did not have the information to be able to categorize children into a specific region or country of origin. Finally, we removed 76 (0.03%) cases in which age of the mother could not be derived from the birth data. This left a confirmed eligible sample of 296 379 births in 26 434 communes with 980 infant deaths, with an average of 11 individuals per commune.

### Statistical methods

To conduct our statistical analyses, we fitted multi-level logit models for binary response variables. In Stata 15.0, we fitted a series of nested mixed-effects logit models using ‘xtmelogit’ with infant mortality as our outcome, with children (level *i*) nested within communes (level *j*). The parameter estimates from the models were exponentiated and interpreted as odds ratios (ORs). In Model 1, we adjusted for parental region of origin only with commune-level effects. In Model 2, we added our vector of individual-level demographic predictors: sex of child, year of birth, age of mother and single vs. multiple birth. In Model 3, we added father’s socio-professional category. In Model 4, we added size of urban unit and deprivation score.

We also conducted two sensitivity analyses. To ensure our findings were robust to different ways of defining the native-born children of immigrants, we redefined them according to the mother’s country of birth only, father’s country of birth only and contingent on both parents being foreign-born. We re-fitted the region models and compared them with the main results. Second, we fitted a complete case analysis, removing the 12% of cases with missing father’s SPC and compared the ORs to the main results. The findings from these sensitivity analyses are documented at the end of the results section and are available online.

## Results

To assess the quality of the data, we compared the number of live births and sex ratio of births in the study period to official estimates from INSEE. Supplementary table S1 confirms an expected sampling rate in the EDP of between 4.1% and 4.5% per year. An average sex ratio of 1.05 in favour of men is identical to INSEE estimates. In Supplementary table S2, the calculated sample infant mortality rate (IMR) of 3.3 indicates only a minor underestimation compared to 3.6 in the INSEE estimates (a ratio of 0.92).

Table 1 presents births, deaths, infant mortality rate per 1000 and the parental origin composition of births in the sample. Births to at least one foreign-born parent comprise 28% of all births; 84% of this share are to parent(s) from a country outside of Europe, with 63% from Africa, consistent with national INSEE estimates.^1^^,^^2^ A sample infant mortality rate (IMR) of 3.31 (3.10–3.52) reflects an average of the lower IMR among children born to two parents born in France [IMR = 2.95 (2.73–3.19)] and higher IMR for children born to at least one foreign-born parent [IMR = 4.23 (3.79–4.69)]. We observe higher IMRs in all groups except Northern and Western Europe and Southern Europe, notably Eastern Europe [IMR = 5.25 (3.67–7.81)], Western Africa [IMR = 7.07 (5.45–9.03)], and Other Sub-Saharan Africa [IMR = 4.72 (3.37–6.43)].

**Table 1:** Births, deaths, infant mortality rates and parental origin composition of births in France, 2008–17

Parental region of origin	Parental origin composition of all births (%)	Parental origin composition of births to immigrants only (%)	Births	Deaths	Infant mortality rate per 1000
All births	–	–	296 379	980	3.31 (3.10–3.52)
Births to two parents born in France	72.1	–	213 766	631	2.95 (2.73–3.19)
Births to at least one foreign-born parent	27.9	–	82 613	349	4.23 (3.79–4.69)
Births to at least one parent from					
Northern and Western Europe	1.5	5.4	4497	13	2.89 (1.54–4.94)
Southern Europe	2.1	7.4	6147	11	1.79 (0.89–3.20)
Eastern Europe	1.5	5.5	4571	24	5.25 (3.67–7.81)
Northern Africa	11.9	42.5	35 109	139	3.96 (3.33–4.68)
Western Africa	3.1	11.0	9052	64	7.07 (5.45–9.03)
Other Sub-Saharan Africa	2.9	10.3	8472	40	4.72 (3.37–6.43)
The Americas	1.1	3.8	3149	17	5.40 (3.15–8.64)
Asia and Oceania	3.9	14.1	11 616	41	3.53 (2.53–4.79)

*Source*: Authors’ calculations based upon Permanent Demographic Sample (EDP), 2008–17.

Table 2 shows the distribution of selected predictors by region of origin. Main trends include the much higher concentration of births to immigrants in large urban areas, the more favourable socioeconomic profiles for Northern and Western Europe and Americas, and less favourable socioeconomic profiles of Northern and Western Africa.

**Table 2:** Distribution of selected background characteristics (%) by parental origin composition in France, 2008–17

Background characteristics	Mainland France	Northern Western Europe	Southern Europe	Eastern Europe	Northern Africa	Western Africa	Other Sub-Saharan Africa	The Americas	Asia and Oceania
Births	213 766	4497	6147	4571	35 109	9052	8472	3149	11 616
Multiple birth									
Yes	3.3	4.1	3.6	3.4	3.4	3.6	3.3	3.4	2.5
Age of mother									
15–19	2.1	1.2	3.0	5.6	1.3	2.6	2.3	1.6	1.5
20–24	13.6	9.2	14.4	15.9	13.6	15.3	13.8	9.5	16.4
25–29	33.9	23.7	26.5	28.4	29.5	30.4	29.3	24.5	30.6
30–34	32.8	35.1	30.3	31.1	30.4	29.1	30.6	34.6	30.6
35–39	14.6	24.2	19.8	15.3	19.3	17.0	18.4	22.5	16.8
40–49	3.0	6.7	6.1	3.8	6.0	5.7	5.7	7.4	4.1
									
Father’s SPC									
Exec and intellectual	12.6	21.1	11.5	15.3	8.2	9.2	10.0	20.7	11.1
Intermediate	23.2	24.1	15.0	14.3	14.0	14.8	17.6	22.6	13.4
Office workers	12.4	10.0	7.4	7.6	10.6	16.3	14.5	10.7	10.8
Manual workers	33.3	23.9	45.7	33.1	42.1	37.2	34.6	27.7	41.2
Farmers	1.8	1.2	0.7	1.2	0.3	0.5	0.7	0.6	0.4
Self-employed	6.7	8.6	7.0	7.1	8.9	5.0	4.9	6.1	9.0
Missing	10.0	11.1	12.7	21.4	16.0	17.1	17.7	11.6	14.1
Size of urban unit									
Rural	26.5	21.2	11.1	7.6	2.8	3.5	4.3	8.7	4.3
Urban, 2–19 999	17.8	14.4	12.7	8.6	7.2	4.8	6.0	8.5	9.5
Urban, 20–199 999	17.1	15.7	17.8	15.7	19.1	12.6	15.7	11.7	19.7
Urban, 200–1 999 999	24.5	26.9	27.4	31.2	37.4	20.1	30.4	23.5	26.0
Paris area	14.1	21.9	31.1	37.0	33.5	59.0	43.5	47.6	40.5
Deprivation index									
Least	19.2	32.1	26.5	27.1	15.6	23.1	21.9	36.5	21.2
Less	20.1	19.2	19.9	19.6	17.8	22.5	21.4	20.9	17.3
Middle	19.9	17.7	19.4	20.9	18.6	16.8	19.2	15.0	16.3
More	20.6	15.9	16.6	14.4	18.6	13.6	16.8	13.0	16.7
Most	20.2	15.1	17.6	18.1	29.4	23.9	20.8	14.6	28.5

*Source*: Authors’ calculations based upon Permanent Demographic Sample (EDP), 2008–17.

**Figure 1:**
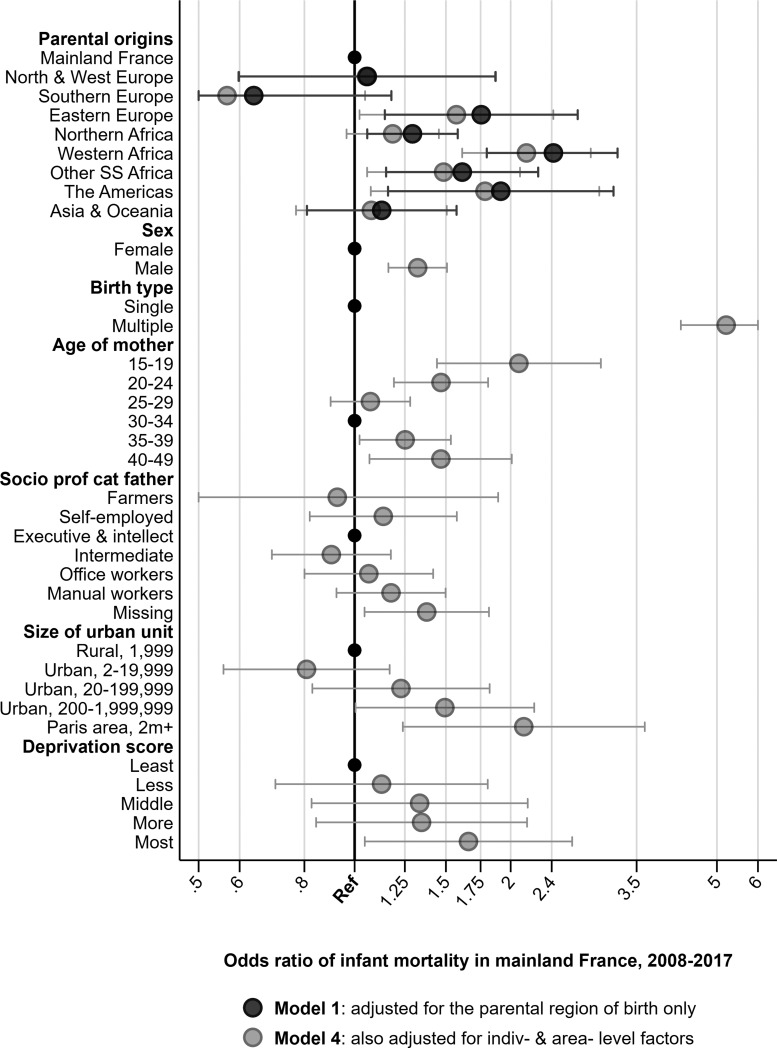
Multi-level logistic regression models of infant mortality among the native-born children of immigrants in France by parental region of origin, 2008–17, unadjusted and adjusted for background characteristics. *Notes*: Lower confidence interval limited at 0.5, upper interval limited at 6, to improve graph readability. *Source*: Authors’ calculations based upon Permanent Demographic Sample (EDP), 2008–17

Figure 1 present the results from the multi-level logit models. We only show the results from Model 1 (which adjusts for parental region of origin only) and Model 4 (which additionally includes all of our individual and area-level predictors). The complete regression tables for Models 1–4 can be found in the Supplementary table S4. In all models, we investigate 980 instances of infant mortality among 296 379 births.

In Model 1, relative to children born to two parents born in mainland France, we observe excess infant mortality among those born to at least one parent from Eastern Europe [OR = 1.76 (1.14–2.69)], Northern Africa [OR = 1.29 (1.06–1.58)], Western Africa [OR = 2.42 (1.82–3.22)], Other Africa [OR = 1.61 (1.15–2.26)] and the Americas [OR = 1.91 (1.16–3.16)]. The size of these excess risks remain similar in Model 2 (with the addition of sex, year, multiple birth and age of mother), are attenuated in Model 3 (with father’s SPC) and again in Model 4 (with urban unit size and deprivation score), but largely persist. Notably, we see Model 4 ORs of: Eastern Europe [OR = 1.57 (1.02–2.42)], Northern Africa [OR = 1.18 (0.96–1.45)], Western Africa [OR = 2.15 (1.61–2.86)], Other Sub-Saharan Africa [OR = 1.49 (1.06–2.09)] and the Americas [OR = 1.78 (1.07–2.96)].

For the other predictors, we found excess infant mortality in Model 4 if the child was male [OR = 1.32 (1.16–1.51)], part of a multiple birth [OR = 5.19 (4.24–6.35)], born to the youngest [OR = 2.08 (1.44–2.99)] or oldest [OR = 3.56 (1.58–8.02)] mothers, an increasing gradient across deprivation score culminating in a sizeable excess in the most deprived communes [OR = 1.66 (1.05–2.63)], and in urban units of 200 000–1,999 999 people [OR = 1.49 (1.00–2.22)] and Paris [OR = 2.12 (1.24–3.63)] consistent with previous work.^4^ There was no clear pattern for father’s SPC. Compared with executive and intellectual positions (the reference group and highest category), intermediate positions have similar ORs of infant mortality. While ORs do rise from intermediate positions through to office workers and down to manual workers, the 95% confidence intervals of all of these estimates overlap. In terms of the share of the excesses explained across models, Supplementary table S6 shows that in Model 4, father’s SPC, urbanicity and FDI together explained 40% of the excess for North Africa and 15–25% of the excess for the other groups. Half of this excess was captured between Model 2 and Model 3, with the rest captured between Model 3 and Model 4.

**Figure 2:**
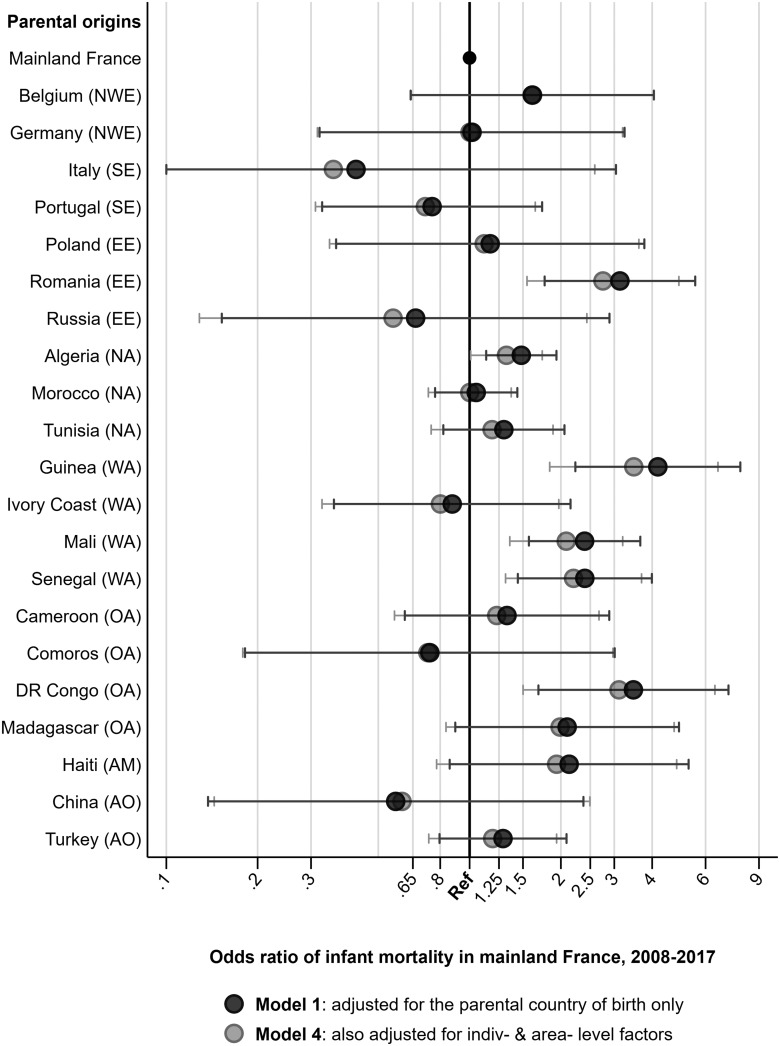
Multi-level logistic regression models of infant mortality among the native-born children of immigrants in France, 2008–17 by parental country of origin, unadjusted and adjusted for background characteristics. *Notes*: Lower confidence interval limited at 0.1, upper interval limited at 9, to improve graph readability. The initials in brackets refer to the broader region of origin from the previous analysis shown in Figure 1; Northern & Western Europe (NWE), Southern Europe (SE), Eastern Europe (EE), Northern Africa (NA), Western Africa (WA), Other Sub-Saharan Africa (OA), The Americas (AM), and Asia & Oceania (AO). *Source*: Authors’ calculations based upon Permanent Demographic Sample (EDP), 2008–17

Figure 2 shows ORs for the top 20 parental countries in terms of the number of births during period 2008–17. Here, we can see which countries are contributing to the excess risks identified in figure 1, the region of origin models. Initially, we observed excess infant mortality among children born to at least one parent who was from Romania [OR = 3.14 (1.77–5.57)], Algeria, [OR = 1.49 (1.14–1.94)], Guinea [OR = 4.19 (2.24–7.85)], Mali [OR = 2.41 (1.58–3.68)], Senegal [OR = 2.41 (1.45–4.00)] and the DR Congo [OR = 3.50 (1.70–7.18)]. In Model 4, we continue to observe elevated infant mortality among children with parental origins from Romania [OR = 2.50 (1.43–4.39)], Algeria, [OR = 1.32 (1.01–1.74)], Guinea [OR = 3.42 (1.81–6.49)], Mali [OR = 1.96 (1.26–3.05)], Senegal [OR = 2.22 (1.32–3.71)] and the DR Congo [OR = 2.87 (1.10–5.48)]. The OR values for Models 1–4 for these country models can be found in Supplementary table S5.

For the sensitivity analyses, Supplementary table S7 shows that we continue to see elevated infant mortality in the same region of origin groups regardless of which definition we use for the native-born children of immigrants. Similarly, a complete case analysis concerning missingness in father’s SPC from Supplementary table S8 shows that we continue to observe elevated infant mortality in the same region of origin groups as shown in figure 1.

## Conclusions

The objectives of this study were to describe initial infant mortality levels among native-born children of immigrants in France and to determine how these levels were affected by adjusting for a range of socio-demographic predictors. To achieve this, we developed a detailed analysis based around a recent period to address the shortcomings of previous research in France.^2^^,^^3^ We documented excess infant mortality among children born to at least one parent from Eastern Europe, Northern Africa, Western Africa, Other Sub-Saharan Africa and the Americas. In the country-specific analysis, we found excess infant mortality levels among countries within these regions, namely Romania (Eastern Europe), Algeria (Northern Africa), Guinea, Mali, Senegal (all Western Africa) and DR Congo (Other Sub-Saharan Africa). In most cases, the excess mortality persisted after having adjusted for individual and area-level predictors.

Our study has many strengths. First, the analysis was based on a large-scale, reliable and representative socio-demographic panel survey. Second, we defined children of immigrants using a stable and reliable indicator—the parental country of birth—providing a great generalizability over time and across national contexts. Third, we provided some of the most granular estimates to date in France or beyond, which included both parental region and country of origin. Fourth, we adopted a novel multi-level approach that considered the role of both individual- and area-level socio-economic factors in variation in infant mortality.

There are also several weaknesses. The sample size limited our ability to investigate trends over time (even if infant mortality levels in France have remained stable over the past decade)^27^, examine causes of death or different forms of infant mortality (e.g. neonatal). We were also limited by small sample sizes, resulting in estimates for smaller country groups (e.g. Italy, Russia and China) with 95% CIs that ranged from sizeable advantages to disadvantages; these ORs should be interpreted with caution. Third, we could not adjust for certain predictors, either due to high levels of missingness (e.g. mother’s SPC and birth order) or because we lacked information (e.g. on health-related variables such as duration of gestation). This lack of more detailed individual-level parental SES predictors (e.g. education level) also limited our ability to capture other aspects of social disadvantage and explain more of the persisting excess mortality.

This study reaffirms that little progress has been made in addressing inequality in infant mortality among the native-born children of immigrants in France during the same period in which significant progress has been made in tackling social inequality.^4–6^ Our findings are consistent with a decade-old study that found elevated infant mortality levels among the native-born children of mothers with Sub-Saharan African nationalities^4^ and a 30-year-old study that found elevated infant mortality among the native-born children of parents with Western African and Northern African nationalities.^6^ Beyond providing recent estimates for France, we have extended the national evidence by documenting the emergence of excess mortality in children with parents from new regions for the first time (e.g. Eastern Europe), by indicating which countries are driving these excesses, and by examining possible socio-demographic causes.

Our data checks found the EDP to be representative of the situation at the national-level. With this in mind, our work represents a valuable addition to the small evidence base on the recent mortality situation of the native-born children of immigrants in France.^28^^,^^29^Our work can help to reignite debate about the lack of progress made in the reduction of this inequality and inform new evidence-based policy. The findings also raise questions about a national citizenship model that advises against the identification of different population sub-groups in France to ensure equal treatment for all. It could be reasoned that the identification of such groups is necessary in order to measure understand, and attempt to address such inequality.

These findings add to the body of European literature identifying elevated infant mortality levels among native-born children of immigrants from non-EU and especially African countries in Belgium,^13^^,^^14^ Denmark,^7^^,^^9^ Italy,^15^ the Netherlands,^18^^,^^19^^,^^30^ Spain,^21^ Sweden,^31^ Switzerland^16^ and the United Kingdom.^12^^,^^20^ We extended this evidence by contributing some of the most detailed and robust findings for a European country lacking recent estimates and adopting a multi-level approach to consider the role of the broader socio-geographic context. Despite the widespread nature of this issue across Europe, there seem to be no specific EU policies in place to address it. The most recent European Perinatal Health Report, for example, made no explicit mention of the children of immigrants.^32^ We call for renewed focus and co-operation across countries in order to address this inequality in early life chances.

Finally, we adopted a multi-level framework in order to consider the role of proximal (the father’s SPC) and distal factors (urbanicity and the deprivation score of the commune) in variation in infant mortality levels. The rationale behind the approach was that variables detailing the wider socio-geographic context might help to capture broader aspects of social disadvantage (and more of the relative mortality excess) that would not be captured by the father’s SPC. While this proved to be the case, large amounts of excess remained in many of the groups. Consequently, we highlight several possible explanations (beyond the specified limitations relating to individual-level SES predictors) that could help to explain the persisting excesses.

In the literature, interpretations relate to the health-seeking behaviours among immigrants and to health-system related factors.^17^ Recent work found that Sub-Saharan African and Northern African mothers were at a greater risks of being overweight or obese prior to pregnancy and having gestational diabetes compared with mothers born in France, factors linked with increased infant mortality.^33^ In Norway and in the Netherlands, non-western immigrants have been found to be less likely to attend antenatal care, leading to poorer detection of complications.^34^ Language barriers may also hamper the interpretation of clinical symptoms and lead to sub-optimal perinatal care, such as inadequate medication and refusal of Caesarean-sections.^35^ This has led some to conclude that, although antenatal care is free in many European countries, some sub-groups might face cultural and lingual barriers to utilizing it effectively.^31^

Additionally, in France, discrimination in the workplace has been found to impact physical and mental health^36^; discrimination has recently been linked with an elevated risk of preterm birth among women in Germany.^37^ For maternal and child health, Sub-Saharan African women were found to have higher risks of stillbirth, preterm births and lower birthweights than French women.^38^ Similarly, the risk of post-partum maternal death was twice as high among foreign women and highest for women from sub-Saharan Africa.^39^^,^^40^ These studies provided evidence that, among women who died, the level of care was more often considered sub-optimal for women with foreign nationality than for women with French nationality.

Further research is needed to understand what specific factors account for these excess infant mortality levels among the native-born children of immigrants in France. Special attention should be given to cultural factors influencing help-seeking behaviours, interactions with the health care system and communication barriers, sub-optimal health care and discrimination. Perinatal audits and detailed analyses of causes of deaths may also provide leads and new avenues for decision makers and public policies.

## Supplementary data

Supplementary data are available at *EURPUB* online.

## Funding

Research reported in this manuscript was supported by the Eunice Kennedy Shriver National Institute of Child Health and Human Development (NICHD) of the National Institutes of Health (NIH) under award number R01HD079475. The content is the responsibility of the authors and does not represent the views of the National Institutes of Health. NIH had no role in the study design, data collection, analysis, interpretation or writing. M.W. had full access to the data and responsibility to submit the paper for publication.

*Conflicts of interest*: None declared.

**Key points**

- Children born in France to at least one parent born in Northern Africa, Western Africa, Other Sub-Saharan Africa, Eastern Europe and the Americas have elevated infant mortality levels.
- These elevated infant mortality levels persist after adjusting for a wide range of individual-level socio-demographic factors and area-level socio-economic and geographical factors.
- At an even more granular level, we find persistent excess infant mortality levels for specific countries in these regional groups, notably Algeria, Mali, Senegal, Guinea, DR Congo and Romania.
- These findings can directly inform public health policy in France, a country that lacks up-to-date and reliable estimates of this nature and enrich wider debates about the health status of immigrants and their children in Europe.

## Supplementary Material

ckaa186_Supplementary_Data

## References

[1] Les naissances en 2018 - Tableaux de séries longues − Les naissances en 2018 | Insee [Internet]. Available at: https://insee.fr/fr/statistiques/4190308?sommaire=4190525 (12 June 2020, date last accessed).

[2] Naissances selon la nationalité et le pays de naissance des parents | Insee [Internet]. Available at: https://www.insee.fr/fr/statistiques/2381382 (12 June 2020, date last accessed).

[3] Oberti M . The French republican model of integration: the theory of cohesion and the practice of exclusion. New Dir Youth Dev 2008;2008:55–74. 18855325 10.1002/yd.273

[4] Niel X . Les Facteurs Explicatifs de la Mortalité Infantile en France et Leur Évolution Récente/Explanatory Factors for Infant Mortality in France and Their Recent Development. Paris: Institut national de la statistique et des études économiques, 2011.

[5] Barbieri M , Toulemon L. Les enfants tous égaux devant la mort? Problèmes d’observation et de mesure des différences sociales de la mortalité infantile en France/Are all children equal before death? Problems observing and measuring social differences in infant mortality in France. In: Histoires de Familles, Histoires Familiales Les Résultats de L’enquête Famille de Ined, 156th edn. Paris: Institut national d’études démographiques, 2005: 407–20.

[6] Quang Chi D . Les inégalités sociales de la mortalité infantile s’estompent/Social inequalities in infant mortality are fading. Estat 1998;314:89–106. 12293991

[7] Villadsen SF , Mortensen LH, Andersen AMN. Ethnic disparity in stillbirth and infant mortality in Denmark 1981–2003. J Epidemiol Community Health 2009 Feb 1;63: 106–12. 18930979 10.1136/jech.2008.078741

[8] Villadsen SF , Sievers E, Andersen A-MN, et al Cross-country variation in stillbirth and neonatal mortality in offspring of Turkish migrants in northern Europe. Eur J Public Health 2010;20:530–5. 20181683 10.1093/eurpub/ckq004

[9] Brehm Christensen M , Fredsted Villadsen S, Weber T, et al Higher rate of serious perinatal events in non-Western women in Denmark. Dan Med J 2016;63:A5197 26931191

[10] Gillet E , Saerens B, Martens G, Cammu H. Fetal and infant health outcomes among immigrant mothers in Flanders, Belgium. Int J Gynecol Obstet 2014;124:128–33. 10.1016/j.ijgo.2013.07.031 24257480

[11] Kinge JM , Kornstad T. Assimilation effects on infant mortality among immigrants in Norway: does maternal source country matter? Demogr Res 2014;31:779–812.

[12] Quayle G . Child and infant mortality in England and Wales - Office for National Statistics [Internet]. 2020. Available at: https://www.ons.gov.uk/peoplepopulationandcommunity/birthsdeathsandmarriages/deaths/bulletins/childhoodinfantandperinatalmortalityinenglandandwales/2018 (12 June 2020, date last accessed).

[13] Racape J , De Spiegelaere M, Alexander S, et al High perinatal mortality rate among immigrants in Brussels. Eur J Public Health 2010;20:536–42. 20478837 10.1093/eurpub/ckq060

[14] Racape J , Schoenborn C, Sow M, et al Are all immigrant mothers really at risk of low birth weight and perinatal mortality? The crucial role of socio-economic status. BMC Pregnancy Childbirth 2016;16:75. 27059448 10.1186/s12884-016-0860-9 PMC4826554

[15] Simeoni S , Frova L, De Curtis M. Inequalities in infant mortality in Italy. Ital J Pediatr 2019;45:11. 30635011 10.1186/s13052-018-0594-6 PMC6330401

[16] Wanner P , Bollini P. The contribution of the foreign population to the high level of infant mortality in Switzerland: a demographic analysis. BMC Pregnancy Childbirth 2017;17:151. 28545468 10.1186/s12884-017-1332-6 PMC5445520

[17] Gissler M , Alexander S, Macfarlane A, et al Stillbirths and infant deaths among migrants in industrialized countries. Acta Obstet Gynecol Scand 2009;88:134 19096947 10.1080/00016340802603805

[18] Troe E-J , Bos V, Deerenberg IM, et al Ethnic differences in total and cause-specific infant mortality in the Netherlands. Paediatr Perinat Epidemiol 2006;20:140–7. 16466432 10.1111/j.1365-3016.2006.00699.x

[19] Troe E-J , Kunst AE, Bos V, et al The effect of age at immigration and generational status of the mother on infant mortality in ethnic minority populations in The Netherlands. Eur J Public Health 2007;17:134–8. 16877451 10.1093/eurpub/ckl108

[20] Bakeo AC . Investigating variations in infant mortality in England and Wales by mother’s country of birth, 1983–2001. Paediatr Perinat Epidemiol 2006;20:127–39. 16466431 10.1111/j.1365-3016.2006.00708.x

[21] Luque-Fernandez MA , Franco M, Gelaye B, et al Unemployment and stillbirth risk among foreign-born and Spanish pregnant women in Spain, 2007–2010. Eur J Epidemiol 2013;28:991–9. 24142267 10.1007/s10654-013-9859-y

[22] Kim D , Saada A. The social determinants of infant mortality and birth outcomes in western developed nations: a cross-country systematic review. Int J Environ Res Public Health 2013;10:2296–335. 23739649 10.3390/ijerph10062296 PMC3717738

[23] Mohamoud YA , Kirby RS, Ehrenthal DB. Poverty, urban-rural classification and term infant mortality: a population-based multilevel analysis. BMC Pregnancy Childbirth 2019;19:40. 30669972 10.1186/s12884-019-2190-1 PMC6343321

[24] Kane JB , Miles G, Yourkavitch J, King K. Neighborhood context and birth outcomes: going beyond neighborhood disadvantage, incorporating affluence. SSM Popul Health 2017;3:699–712. 29349258 10.1016/j.ssmph.2017.08.003 PMC5769105

[25] Windenberger F , Rican S, Jougla E, Rey G. Spatiotemporal association between deprivation and mortality: trends in France during the nineties. Eur J Public Health 2012;22:347–53. 21459841 10.1093/eurpub/ckr029 PMC3904423

[26] Rey G , Jougla E, Fouillet A, Hémon D. Ecological association between a deprivation index and mortality in France over the period 1997–2001: variations with spatial scale, degree of urbanicity, age, gender and cause of death. BMC Public Health 2009;9:33. 19161613 10.1186/1471-2458-9-33 PMC2637240

[27] Papon S . La mortalité infantile est stable depuis dix ans après des décennies de baisse—Insee Focus—117 [Internet]. 2018. Available at: https://www.insee.fr/fr/statistiques/3560308 (17 June 2020, date last accessed).

[28] Guillot M , Khlat M, Wallace M. Adult mortality among second-generation immigrants in France: results from a nationally representative record linkage study. Demogr Res 2019;40:1603–44. 10.4054/demres.2019.40.54 PMC8114944 33986627

[29] Khlat M , Wallace M, Guillot M. Divergent mortality patterns for second generation men of North-African and South-European origin in France: role of labour force participation. SSM Popul Health 2019;9:100447. 31497637 10.1016/j.ssmph.2019.100447 PMC6718938

[30] Schulpen TWJ , van Wieringen JCM, van Brummen PJ, et al Infant mortality, ethnicity, and genetically determined disorders in The Netherlands. Eur J Public Health 2006;16:290–3. 10.1093/eurpub/cki201 16207723

[31] Essén B , Hanson BS, Östergren P-O, et al Increased perinatal mortality among sub-Saharan immigrants in a city-population in Sweden. Acta Obstet Gynecol Scand 2000;79:737–43. 10993096

[32] Euro-Peristat Project. European Perinatal Health Report. Core indicators of the health and care of pregnant women and babies in Europe in 2015 [Internet]. 2018. Available at: https://www.europeristat.com (16 June 2020, date last accessed).

[33] El-Khoury Lesueur F , Sutter-Dallay A-L, Panico L, et al The perinatal health of immigrant women in France: a nationally representative study. Int J Public Health 2018;63:1027–36. 30097678 10.1007/s00038-018-1146-y

[34] Alderliesten ME , Vrijkotte TGM, Wal MVD, Bonsel GJ. Late start of antenatal care among ethnic minorities in a large cohort of pregnant women. BJOG 2007;114:1232–9. 17655734 10.1111/j.1471-0528.2007.01438.x

[35] Essén B , Bödker B, Sjöberg N-O, et al Are some perinatal deaths in immigrant groups linked to suboptimal perinatal care services? BJOG 2002;109:677 12118647

[36] Cognet M , Hamel C, Moisy M. Santé des migrants en France : l’effet des discriminations liées à l’origine et au sexe. Remi 2012;28:11–34.

[37] Scholaske L , Brose A, Spallek J, Entringer S. Perceived discrimination and risk of preterm birth among Turkish immigrant women in Germany. Soc Sci Med 2019;236:112427. 31352314 10.1016/j.socscimed.2019.112427 PMC7327293

[38] Saurel-Cubizolles MJ , Saucedo M, Drewniak N, et al Santé périnatale des femmes étrangères en France. Bulletin Epidémiologique Hebdomadaire 2012; 2-3-4:30–4.

[39] Philibert M , Deneux‐Tharaux C, Bouvier‐Colle M-H. Can excess maternal mortality among women of foreign nationality be explained by suboptimal obstetric care? BJOG 2008;115:1411–8. 18823490 10.1111/j.1471-0528.2008.01860.x

[40] Saucedo M , Deneux‐Tharaux C, Bouvier‐Colle MH. Epidémiologie des morts maternelles en France, 2001-2006. Bulletin Epidémiologique Hebdomadaire 2010;2-3:10–4.

