# Supplementary material for: Infant mortality among native-born children of immigrants in France, 2008–17: results from a socio-demographic panel survey
Source: Eur J Public Health. 2020 Nov 30;31(2):326–33. doi: 10.1093/eurpub/ckaa186 (PMC8071600; doi:10.1093/eurpub/ckaa186)
Supplement: ckaa186_Supplementary_Data [file ckaa186_supplementary_data.docx]

**Supplementary materials**

**Table S1.** The sex ratio of births in the Permanent Demographic Sample compared to national INSEE estimates and sex-specific sampling rate of the EDP (i.e. EDP / INSEE).

| **Birth year** | **EDP** | | | **National estimates** | | | **Sampling rate** | |
| --- | --- | --- | --- | --- | --- | --- | --- | --- |
|  | Males | Females | Sex Ratio | Males | Females | Sex Ratio | Males | Females |
| 2008 | 18,258 | 17,426 | **1·05** | 406,784 | 389,260 | **1·05** | 4·5% | 4·5% |
| 2009 | 17,459 | 16,474 | **1·06** | 405,902 | 387,518 | **1·05** | 4·3% | 4·3% |
| 2010 | 17,222 | 16,348 | **1·05** | 410,140 | 392,084 | **1·05** | 4·2% | 4·2% |
| 2011 | 16,980 | 16,062 | **1·06** | 405,206 | 387,790 | **1·04** | 4·2% | 4·1% |
| 2012 | 17,902 | 16,797 | **1·07** | 404,774 | 385,516 | **1·05** | 4·4% | 4·4% |
| 2013 | 17,748 | 17,060 | **1·04** | 400,149 | 381,472 | **1·05** | 4·4% | 4·5% |
| 2014 | 17,623 | 16,739 | **1·05** | 399,284 | 381,883 | **1·05** | 4·4% | 4·4% |
| 2015 | 16,339 | 15,793 | **1·03** | 389,181 | 371,240 | **1·05** | 4·2% | 4·3% |
| 2016 | 15,729 | 15,303 | **1·03** | 381,310 | 363,387 | **1·05** | 4·1% | 4·2% |

*Source: authors’ calculations based upon Permanent Demographic Sample (EDP), 2008-2017 and INSEE national estimates.*

**Table S2.** Infant mortality in the EDP versus infant mortality from national estimates.

| **Year** | **IMR per 1,000 live births, INSEE** | **IMR per 1,000 live births,**  **EDP** | **Ratio** |
| --- | --- | --- | --- |
| 2008 | 3·8 | 3·8 | 1·00 |
| 2009 | 3·9 | 3·3 | 0·85 |
| 2010 | 3·6 | 3·2 | 0·89 |
| 2011 | 3·5 | 3·6 | 1·02 |
| 2012 | 3·5 | 3·3 | 0·93 |
| 2013 | 3·6 | 3·1 | 0·94 |
| 2014 | 3·5 | 3·0 | 0·86 |
| 2015 | 3·6 | 3·9 | 1·03 |
| 2016 | 3·7 | 3·2 | 0·86 |
| 2008-2016 | 3·6 | 3·3 | 0·92 |

*Source: authors’ calculations based upon Permanent Demographic Sample (EDP), 2008-2017 and Insee national estimates.*

**Table S3.** Origin country composition of the regional groups; top ten countries and relative share.

| **UN region** | **Top maternal origin countries (ranked with relative %)** | | | |
| --- | --- | --- | --- | --- |
|  | **1st** | **2nd** | **3rd** | **4th-10th** |
| **Northern &  Western Europe** | Germany  (25%) | Belgium  (25%) | United Kingdom  (18%) | Switzerland, Netherlands, Sweden, Lithuania, Ireland, Australia, Latvia (24%) |
| **Southern  Europe** | Portugal  (48%) | Italy  (14%) | Yugoslavia  (13%) | Spain, Albania, Greece, Macedonia, Croatia, Serbia, Slovenia (24%) |
| **Eastern  Europe** | Romania  (32%) | Russia  (19%) | Poland  (18%) | Bulgaria, Moldova, Ukraine, Bosnia, Hungary, Belarus, Czech Rep (21%) |
| **Northern  Africa** | Algeria  (43%) | Morocco  (40%) | Tunisia  (15%) | Egypt, Libya (2%) |
| **Other SS  Africa** | Congo  (22%) | Cameroon  (19%) | Comoros  (12%) | Madagascar, DR Congo, Gabon, Angola, CAR, Mauritius, Chad (42%) |
| **Western  Africa** | Senegal  (25%) | Ivory Coast  (20%) | Mali  (20%) | Guinea, Nigeria, Cape Verde, Mauritiana, Togo, Benin, Burkina Faso (33%) |
| **The  Americas** | Haiti  (22%) | Brazil  (15%) | USA  (11%) | Colombia, Mexico, Canada, Peru, Chile, Argentina, Venezuela (36%) |
| **Asia &  Oceania** | Turkey  (38%) | China  (9%) | Sri Lanka  (7%) | Vietnam, India, Cambodia, Armenia, Lebanon, Pakistan, Thailand (22%) |

*Source: authors’ calculations based upon Permanent Demographic Sample (EDP), 2008-2017*

**Table S4.** Full regression tables, mothers from UN-defined origin regions (main Figure 1).

*Source: authors’ calculations based upon Permanent Demographic Sample (EDP), 2008-2017*

Notes: values significant to p<0.01 **, p<0.05 *, and p <0.10 +.

**Table S4.** (cont.)

*Source: authors’ calculations based upon Permanent Demographic Sample (EDP), 2008-2017*

Notes: values significant to p<0.01 **, p<0.05 *, and p <0.10 +.

**Table S5.** Odds ratios for top twenty origin countries (main Figure 2).

*Source: authors’ calculations based upon Permanent Demographic Sample (EDP), 2008-2017*

Notes:

1. Model 1 adjusts for parental region of origin only; Model 2 then adjusts for sex of child, year of birth, age of mother, and single vs. multiple birth; Model 3 further adjusts for the father’s socio-professional category; Model 4 finally adjusts for the size or urban unit and deprivation score of the mother’s commune of residence.
2. We do not show the values in the predictors because they are practically identical to those presented in Figure 1 and shown in Table S4.
3. Values significant to p<0.01 **, p<0.05 *, and p <0.10 +.

**Table S6.** Proportion of excess infant mortality explained, where excess is observed, between models.

*Source: authors’ calculations based upon Permanent Demographic Sample (EDP), 2008-2017*

Notes:

1. Model 1 adjusts for parental region of origin only; Model 2 then adjusts for sex of child, year of birth, age of mother, and single vs. multiple birth; Model 3 further adjusts for the father’s socio-professional category; Model 4 finally adjusts for the size or urban unit and deprivation score of the mother’s commune of residence.
2. We do not show the values in the predictors because they are practically identical to those presented in Figure 1 and shown in Table S4.
3. Values significant to p<0.01 **, p<0.05 *, and p <0.10 +.

**Table S7.** Comparisons of ORs of parental region of origin according to whether at least one parent is foreign-born or only the mother is foreign-born.

*Source: authors’ calculations based upon Permanent Demographic Sample (EDP), 2008-2017*

Notes:

1. Model 1 adjusts for parental region of origin only; Model 2 then adjusts for sex of child, year of birth, age of mother, and single vs. multiple birth; Model 3 further adjusts for the father’s socio-professional category; Model 4 finally adjusts for the size or urban unit and deprivation score of the mother’s commune of residence.
2. We do not show the values in the predictors because they are practically identical to those presented in Figure 1 and shown in Table S4.
3. Values significant to p<0.01 **, p<0.05 *, and p <0.10 +.

**Table S8.** Comparisons of ORs for a complete case analysis, excluding cases with missing SCP for the father and the primary analysis in the paper, which keeps these individuals with father’s SCP as missing.

*Source: authors’ calculations based upon Permanent Demographic Sample (EDP), 2008-2017*

Notes:

1. Model 1 adjusts for parental region of origin only; Model 2 then adjusts for sex of child, year of birth, age of mother, and single vs. multiple birth; Model 3 further adjusts for the father’s socio-professional category; Model 4 finally adjusts for the size or urban unit and deprivation score of the mother’s commune of residence.
2. We do not show the values in the predictors because they are practically identical to those presented in Figure 1 and shown in Table S4.
3. Values significant to p<0.01 **, p<0.05 *, and p <0.10 +.
